# Identification of middle cerebral artery stenosis in transcranial Doppler using a modified VGG-16

**DOI:** 10.3389/fneur.2024.1394435

**Published:** 2024-10-16

**Authors:** Dong Xu, Hao Li, Fanghui Su, Sizheng Qiu, Huixia Tong, Meifeng Huang, Jianzhong Yao

**Affiliations:** ^1^Department of Neuroelectrophysiology, Anyang People's Hospital, Anyang, China; ^2^Shenzhen Institute for Advanced Study, University of Electronic Science and Technology of China, Shenzhen, China

**Keywords:** transcranial Doppler, ICAS, deep learning, stroke, screening

## Abstract

**Objectives:**

The diagnosis of intracranial atherosclerotic stenosis (ICAS) is of great significance for the prevention of stroke. Deep learning (DL)-based artificial intelligence techniques may aid in the diagnosis. The study aimed to identify ICAS in the middle cerebral artery (MCA) based on a modified DL model.

**Methods:**

This retrospective study included two datasets. Dataset1 consisted of 3,068 transcranial Doppler (TCD) images of the MCA from 1,729 patients, which were assessed as normal or stenosis by three physicians with varying levels of experience, in conjunction with other medical imaging data. The data were used to improve and train the VGG16 models. Dataset2 consisted of TCD images of 90 people who underwent physical examination, which were used to verify the robustness of the model and compare the consistency between the model and human physicians.

**Results:**

The accuracy, precision, specificity, sensitivity, and area under curve (AUC) of the best model VGG16 + Squeeze-and-Excitation (SE) + skip connection (SC) on dataset1 reached 85.67 ± 0.43(%),87.23 ± 1.17(%),87.73 ± 1.47(%),83.60 ± 1.60(%), and 0.857 ± 0.004, while those of dataset2 were 93.70 ± 2.80(%),62.65 ± 11.27(%),93.00 ± 3.11(%),100.00 ± 0.00(%), and 0.965 ± 0.016. The kappa coefficient showed that it reached the recognition level of senior doctors.

**Conclusion:**

The improved DL model has a good diagnostic effect for MCV stenosis in TCD images and is expected to help in ICAS screening.

## 1 Introduction

Intracranial atherosclerotic stenosis (ICAS) is one of the important risk factors for stroke. In recent years, a growing number of studies have shown that the middle cerebral artery (MCA) is the most susceptible intracranial artery to stenosis, which significantly increases the risk of transient ischemic attacks (TIAs) and stroke recurrence (1). Asymptomatic ICAS is increasingly recognized as a risk factor for silent cerebral infarction and dementia, and ICAS leads to a greater risk with age, thus greatly increasing the medical burden of cerebral infarction caused by ICAS (2).

Therefore, it is very important to identify ICAS. Clinically, doctors use different detection methods to determine whether a patient has MCA stenosis, including transcranial Doppler (TCD), magnetic resonance imaging (MRI), computed tomography (CT), and digital subtraction angiography (DSA). TCD is a relatively simple, non-invasive, and cost-effective method for the detection of ICAS. Compared with DSA, CTA, and MRA, TCD can provide examination results quickly within minutes, providing point-of-care examination for people who cannot tolerate traditional neuroimaging due to its portability (3). In addition, MRA remains expensive and impractical as a cerebrovascular screening method in primary hospitals and some rural areas of certain developing countries. Therefore, TCD is extremely appropriate and efficient as a screening tool for intracranial vascular lesions (4). However, despite the advantages of TCD, the accuracy of TCD interpretation is highly dependent on the experience of the examining physician, and the accurate identification of lesion images requires a lot of time and training. According to findings of the World Health Organization, with the increasing shortage of doctors, there may be a shortage of nearly 13 million health workers worldwide by 2035 (5). This also means that there will be a serious shortage of health workers with relevant TCD image diagnosis experience in the future. In recent years, the emergence of sophisticated computer-aided diagnosis tools, represented by Deep Learning (DL), may alleviate this problem.

DL is an artificial intelligence algorithm that has emerged in recent years and has been proven to have high accuracy in many medical image recognition and diagnosis tasks (6). DL extracts features from different medical images through a neural network structure to train itself and make diagnoses, such as distinguishing between benign and malignant tumors (7), identifying abnormal ECG (8), assisting in pathological diagnosis (9), and classification (10). Currently, there are few studies on the application of artificial intelligence, especially the DL algorithm, in diagnosing TCD images. The evaluation of the effectiveness of the improved model structure and the comparison with human doctors have not been studied in depth. Therefore, this study aimed to explore the feasibility of DL in TCD image recognition and diagnosis. The model was tested using TCD images of the MCA and improved based on the common DL model previously used for TCD images. Finally, the trained model was compared with doctors who have rich diagnostic experience.

Organization: This article is divided into four parts. The first part is the introduction. The second part covers the methods and materials of the research, with a focus on the construction of the deep learning model. The third part presents the experimental results and data. The fourth part encompasses the discussion and summary.

## 2 Methods

This retrospective cross-sectional study was approved by the Ethics Committee of Anyang People’s Hospital (KS-2023-04-10), and the consent forms were waived.

### 2.1 Deep learning background

Deep learning is an algorithm that has gradually gained attention from researchers in various fields in recent years. The primary approach involves identifying the type of task and then collecting a large amount of relevant data. Through feature learning from a large number of samples, a powerful feature extraction model can be developed. Then, subsequently tailored to the specific demands of tasks, such as image object segmentation, object detection, and object classification, specific functions can be completed. In recent years, it has been applied as a powerful auxiliary tool in various medical and clinical processes (11, 12).

VGG16 is a classic deep learning feature extraction network that consists of 13 convolutional layers and three fully connected layers, which is why it is named VGG16. Specifically, all the convolutional layers of the VGG network use 3 × 3 convolution kernels, followed by a rectified linear unit (ReLU) activation function for non-linear mapping. Its MaxPooling layer uses a 2 × 2 convolution kernel with a stride of 2, which can reduce the width and height of the feature map by half while maintaining the same number of channels. After five feature extraction blocks, there are three fully connected layers, with the feature channel output of the first two layers being 4,096 and the output of the last layer being 1,000. Finally, a softmax layer is used to output the final predicted probability values, with the class corresponding to the highest value being the predicted class of the model.

### 2.2 Data acquisition

This study included 1,729 patients who visited our hospital from July 2021 to September 2022. All patients underwent TCD and other cerebrovascular imaging examinations (CTA, DSA, or MRA), and the diagnosis of either stenosis or normal conditions in the middle cerebral arteries was confirmed through comprehensive imaging results. The TCD examinations were conducted by three physicians with 1–12 years of experience in TCD procedures and interpretation. The machines used for the examinations were the MVU-6300 and EMS-9A. The TCD spectral images of the bilateral middle cerebral arteries, recorded using a 2 MHz pulse probe, were collected from the examination results. All low-quality images or those mistakenly stored due to improper handling were removed, and the remaining clear and readable images were compiled into a dataset. As we intended to utilize the constructed DL model for ICAS screening in the future, it would have been more aligned with real-world scenarios to use data from a population undergoing routine medical check-ups. However, it is worth noting that the dataset from the population undergoing routine medical check-ups is characterized by fewer cases of severe ICAS and some complex situations. Therefore, we decided to train the model using data from hospitalized patients and collected a portion of TCD data from a population undergoing routine medical check-ups for validation purposes.

Among them, the TCD data from hospitalized patients examined using the MVU-6300 were categorized as dataset1, while the TCD data from the population undergoing routine medical check-ups examined using the EMS-9A were categorized as dataset2. All samples in dataset1 were labeled by two TCD-experienced physicians as either stenosis or non-stenosis. In case of disagreement, a third physician with more extensive TCD experience, along with information from CTA, DSA, or MRA, made the final judgment and labeled the samples. Dataset1 was divided into a 70% training set (2,146 images), a 10% validation set (308 images), and a 20% test set (614 images). Dataset2, a small dataset derived from the population undergoing routine medical check-ups, consisted of 90 images and was solely used for validating the performance of the model on an external dataset. In addition, dataset2 was annotated by another TCD physician, who was independent of the previous three physicians, to ensure blinding of the data. This was done for the subsequent assessment of the consistency between the physicians and the DL model, thereby evaluating their performance. Finally, two physicians with varying years of TCD experience used dataset2 for judgment, and the results were compared with those of the DL models. To the best of our knowledge, no such comparison has been conducted. This represents another novel aspect of this study, which may help the DL model to be used for large-scale screening of cerebral vascular stenosis in the future.

### 2.3 Experimental environment

The experiments in this study were performed on Ubuntu20.04, using the Pytorch deep learning framework. Furthermore, the hardware configuration used was an Intel Core i7 12,700 CPU with a 2.1G main frequency, 64G memory, an NVIDIA GeForce RTX 3070Ti GPU, and a 1 T mechanical hard disk. The algorithm was written in Python programming language, and the required configuration environment for the algorithm was conda22.9.0, python3.6.13, opencv3.4.3, pytorch1.10.2, torchvision0.11.3, cuda11.3.0, cudnn8.6.0, matplotlib3.3.4, pandas1.1.5, and numpy1.19.5.

### 2.4 Deep learning model and training

The loss function used in this study was LabelSmoothingCrossEntropy, a regularization method that can prevent overconfident predictions of the model during the training process in classification tasks and improve the generalization ability. Its formula is expressed as follows Equations 1, 2:


(1) $${\hat{y}}_{i}=y_{\mathrm{hot}}\left(1-\alpha \right)+\frac{\alpha }{K}$$

(2) $${\hat{y}}_{i}=\{\begin{array}{c} \frac{\alpha }{K'}\;i\neq \mathrm{target}\\ 1-\alpha +\frac{\alpha }{K}\;i=\mathrm{target} \end{array}$$

Where i denotes the sample number, target refers to the current category, ${\hat{y}}_{i}$ denotes the updated label vector, and $y_{\mathrm{hot}}$ denotes the one-hot encoded label vector. *α* is a small hyperparameter (generally 0.1), and K is the total number of categories. The loss function formula (Equation 3) used in this paper is as follows:


(3) $$\mathrm{Loss}=-\overset{K}{\underset{i=0}{\sum }}\hat{y}\log\left(p\left(x_{i}\right)\right)*\left(1-\alpha \right)-\frac{1}{K}\overset{K}{\underset{i=1}{\sum }}\log\left(x_{i}\right)*\alpha$$

VGG16 is composed of five convolutional blocks followed by three fully connected layers. We denote the five convolutional blocks as the Feature Extraction Layer. Firstly, a single image is passed through the Feature Extraction Layer. We denote the input image as $x_{i}$ and the output of the Feature Extraction Layer as out_f. Then, a 7 × 7 convolution is used for max pooling, followed by a fully connected layer and rectified linear units (ReLU) as transition layers, which are repeated three times. In the end, a softmax layer is performed to obtain the prediction result.

out_f = Feature Extraction Layer( $x_{i}$ )

out_1 = MaxPooling(out_f)

out_2 = ReLU(FC(out_1))

out_3 = ReLU(FC(out_2))

out_4 = ReLU(FC(out_3))

final_output = Softmax(out_4)

To achieve higher classification accuracy, we made improvements to the VGG16 model. This article includes the two main improvements.

Firstly, the VGG16 model does not consider the importance of different feature channels when learning input image features. To address this issue, we added a Squeeze-and-Excitation (SE) module based on attention mechanism (13). During the network training phase, the SE module can focus on useful features and suppress useless features, allowing the model to learn the importance of each feature channel.

In the Squeeze stage, the formal expression is (Equation 4) as follows:


(4) $$k_{c}=F_{\mathrm{squeeze}}\left(m_{c}\right)=\frac{1}{H\times W}\overset{H}{\underset{i=1}{\sum }}\overset{W}{\underset{j=1}{\sum }}m_{c}\left(i,j\right)$$

This obtains channel-wise statistical information by using global average pooling.

$F_{\mathrm{squeeze}}\left(\cdot \right)$ denotes the squeeze operation, m represents the feature map, $m_{c}$ denotes the c-th channel of the feature map, and $k_{c}$ is the average value of the feature map in the c-th channel.

In the Excitation stage, the formal expression (Equations 5, 6) is as follows:


(5) $$e=F_{\mathrm{excitation}}\left(k,W\right)=\mathrm{sigmoid}\left(g\left(k,W\right)\right)=\mathrm{sigmoid}\left(W_{2}\mathrm{ReLU}\left(W_{1}k\right)\right)$$

(6) $${\tilde{x}}_{C}=F_{Re‐\mathrm{weight}}=\left(m_{c},e_{c}\right)=e_{c}m_{c}$$

Where $F_{\mathrm{excitation}}\left(\cdot \right)$ represents the excitation operation, $W_{1}\in R^{\frac{C}{r}\times C}$ and $W_{2}\in R^{C\times \frac{C}{r}}$ represent the weight matrices of two fully connected layers, and r represents the number of hidden nodes in the intermediate layer. $F_{Re-\mathrm{weight}}\left(\cdot \right)$ represents the operation of adjusting channel weights, $\tilde{X}=\left[\tilde{x_{1}},\tilde{x_{2}},\ldots ,\tilde{x_{C}}\right]$ , where ${\tilde{x}}_{c}$ is a feature map of a feature channel of $\tilde{X},\mathrm{and}\;e_{c}$ is a scalar value in the gating unit e .

Secondly, the VGG16 model uses linear feature extraction, where each convolutional layer uses the features extracted from the previous layer as input. It does not incorporate the element of multi-scale feature fusion, which can lead to incomplete feature extraction of the input image. To address this issue, we added two skip connections (SCs) to implement multi-scale feature fusion. These skip connections can effectively transmit features of different scales to multiple layers after convolution and rescaling, enabling the complete fusion of different feature scales.

In deep learning, SCs are techniques that directly connect non-adjacent layers by skipping one or more layers. These skip connections can effectively alleviate the vanishing and exploding gradients in deep neural networks and facilitate the propagation of information throughout the network. Common SC methods include residual connections and dense connections. In our study, we adopted an approach similar to residual connections, establishing an SC between the input and the first max-pooling layer. This allowed the input to be fed into the next layer along with the results that were processed by Conv+SE + BN + ReLU and max pooling. Similarly, another SC was established between the second and third max-pooling layers, adding more pathways for information propagation.

**Figure 1:**
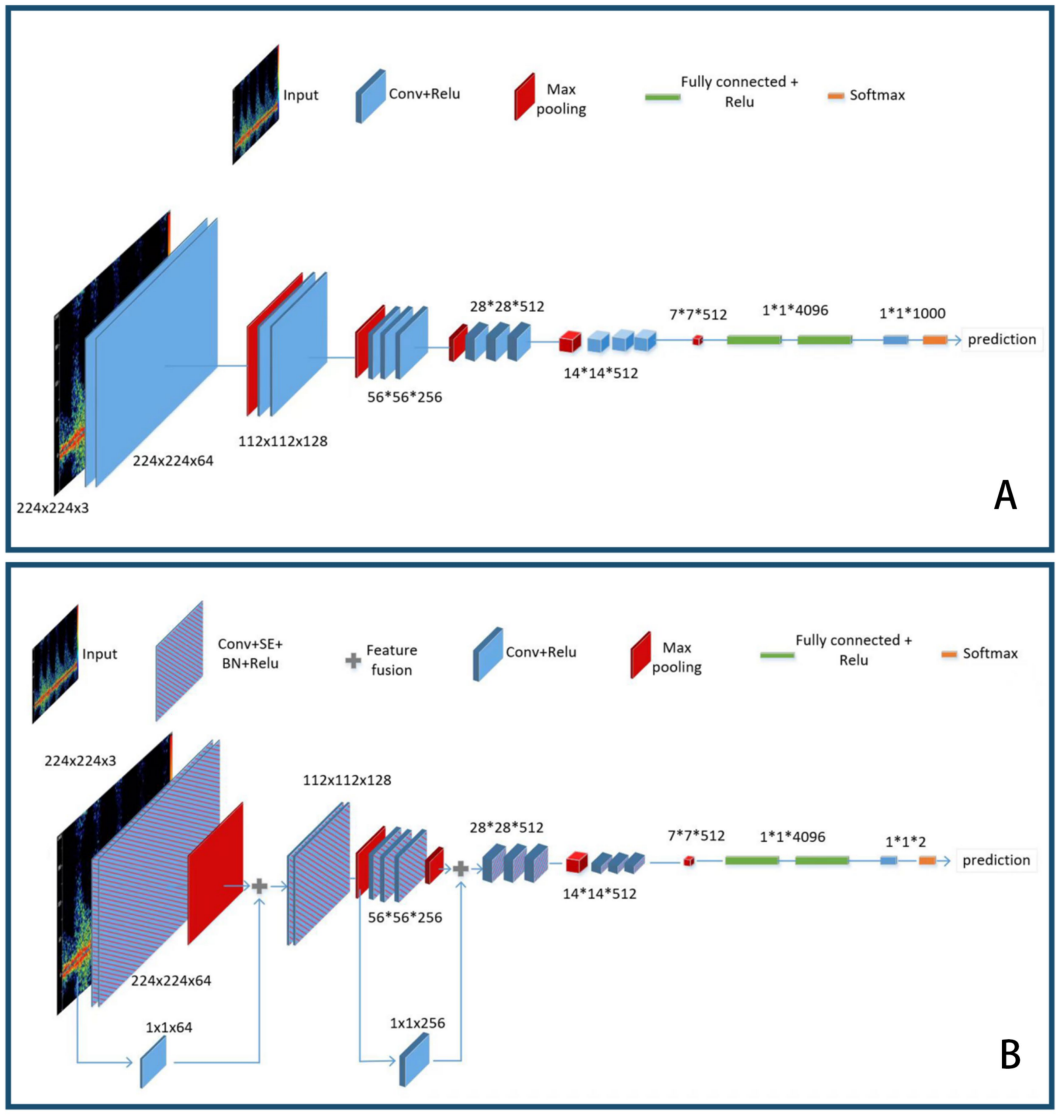
(A) VGG16 network structure. (B) Modified VGG16 network structure.

Overall, SCs increase the direct propagation paths for gradients, ensuring that gradients can be smoothly propagated to shallower layers, which helps accelerate the training process of the network. In addition, adding skip connections can capture more diverse features of the target objects, enhancing the network’s representation ability and thus improving model performance. See Figure 1B for the details of the skip connection, which was another important improvement to the VGG network structure.

The improved VGG16 consists of five convolutional blocks and two skip connection layers, followed by three fully connected layers. In this model, the original Conv+Relu block has been replaced by Conv+SE + BN + Relu. Each convolution block containing the SE block is marked as block i_with_SE (where i denotes the index of the block). The feature fusion from the input image(marked as $x_{i}$ ) to the first max pooling, which uses 1 × 1 convolution(conv1d), is marked as ff1. The feature fusion between the second max pooling and the third max pooling is marked as ff 2. After that, similar operations are performed. Once the 5-layer convolution block is completed, we obtain the feature map. After one max pooling and three fully connected layers +ReLU, finally, the final prediction result is obtained through a softmax layer.

out1 = block1_with_SE( $x_{i}$ )

fusion1 = ff1(conv1d( $x_{i}$ ),out1)

out2 = block2_with_SE(fusion1)

out3 = block3_with_SE(out2)

fusion2 = ff2(conv1d(out2),out3)

out4 = block4_with_SE(fusion2)

out5 = block5_with_SE(out4)

out_max = MaxPooling(out5)

out_relu1 = ReLU(FC(out_max))

out_relu2 = ReLU(FC(out_relu1))

out_relu3 = ReLU(FC(out_relu2))

final_output = Softmax(out_relu3)

The schematic diagram of the VGG16 model before and after the improvement is shown in Figure 1.

### 2.5 Statistical analysis

We used accuracy, precision, specificity, sensitivity, and area under curve (AUC) to evaluate model performance. Inter-observer variability among the different models and between the models and the TCD physicians was calculated using kappa coefficients.

## 3 Results

We used the confusion matrix, a commonly used concept in machine learning and statistics, to evaluate the performance of the classification model. The confusion matrix is composed of true positive (TP), false positive (FP), false negative (FN), and true negative (TN).

Based on the confusion matrix, we calculated the following metrics to compare our model’s performance across different aspects.


$$\mathrm{Accuracy}=\frac{\left(TP+TN\right)}{\left(TP+FP+TN+FN\right)}$$

$$\mathrm{Precision}=\frac{\left(TP\right)}{\left(TP+FP\right)}$$

$$\mathrm{Specificity}=\frac{\left(TN\right)}{\left(FP+TN\right)}$$

$$\mathrm{Sensitivity}=\frac{\left(TP\right)}{\left(TP+FN\right)}$$

**Figure 2:**
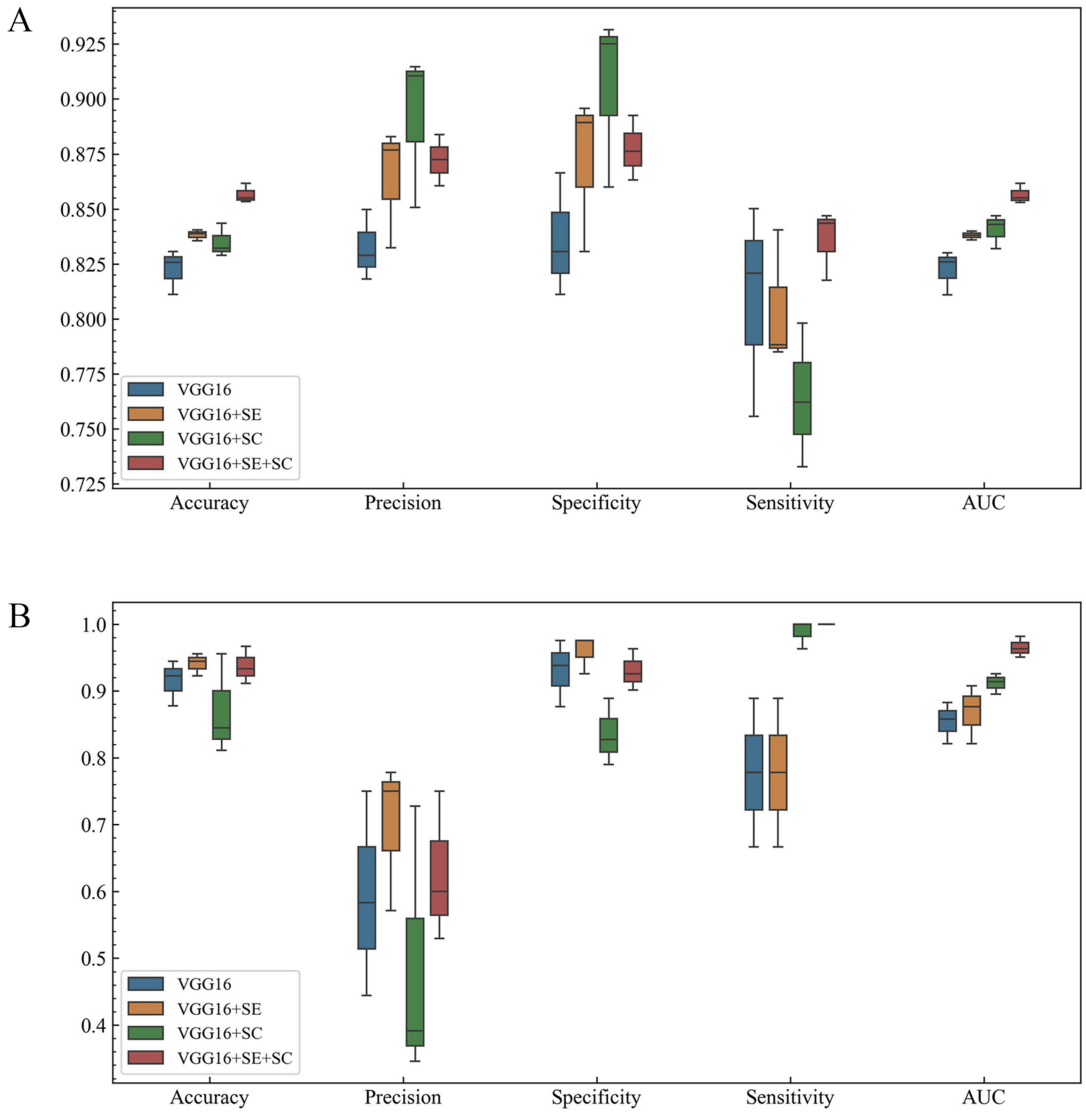
The performance of the algorithmic models. (A) Each algorithmic model on dataset1. (B) Each algorithmic model on dataset2. It can be observed that VGG16 + SE + SC achieved a higher mean and a smaller standard deviation.

We have displayed the performance of the different structural DL models on dataset1 in Table 1 and visually presented the strengths and weaknesses of model performance across the various metrics in Figure 2A. On the test of dataset1, the VGG16 + SE + SC model achieved the best performance with an AUC of 0.857 ± 0.004. Its accuracy (%) and sensitivity (%) also exhibited the best scores of 85.67 ± 0.43 and 83.60 ± 1.60, respectively. In addition, we can intuitively see from Figure 2 that the VGG16 + SE + SC model exhibited a smaller range of standard deviation across all evaluation standards, indicating its robustness.

**Table 1:** Performance of the different models on dataset1.

Dataset1					
Model	Accuracy (%)	Precision (%)	Specificity (%)	Sensitivity (%)	AUC
VGG16	82.24 ± 1.01	83.23 ± 1.61	83.60 ± 2.80	80.89 ± 4.84	0.822 ± 0.010
VGG16 + SE	83.82 ± 0.25	86.40 ± 2.76	87.19 ± 3.59	80.46 ± 3.11	0.838 ± 0.002
VGG16 + SC	83.49 ± 0.77	89.19 ± 3.58	90.55 ± 3.97	76.44 ± 3.26	0.841 ± 0.008
VGG16 + SE + SC	85.67 ± 0.43	87.23 ± 1.17	87.73 ± 1.47	83.60 ± 1.60	0.857 ± 0.004

Next, we utilized dataset2 to evaluate the effectiveness of our model in screening for cerebral vascular stenosis in the physical examination population, employing the same indicators as in dataset1 to evaluate model performance. The results are shown in Figure 2B. It is evident that the improved model still demonstrated strong performance on the new dataset, with an AUC of 0.965 ± 0.016, indicating an extremely high diagnostic standard.

**Figure 3:**
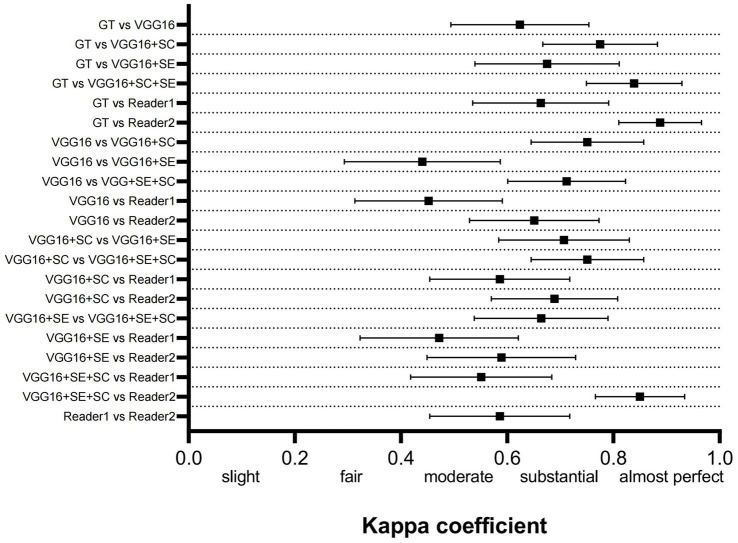
Comparison of the consistency between the 3 DL models, GT, and human physicians. GT, ground truth.

Figure 3 shows the kappa coefficient to assess the agreement among the various models, between the models and the ground truth (GT), and between the models and the physicians. The classification of the kappa coefficient is as follows: if k ≤ 0.2, it is considered slight; if 0.2 < k ≤ 0.4, it is considered fair; if 0.4 < k ≤ 0.6, it is considered moderate; if 0.6 ≤ k < 0.8, it is considered substantial; and if k ≥ 0.8, it is considered almost perfect (14). We have visually presented the results of the consistency analysis in Figure 3. The results revealed that the improved model had strong consistency with both the highly experienced physician (reader2) and the GT. The high consistency between reader2 and the GT indirectly suggests that our improved model approaches the TCD imaging diagnostic level of a highly experienced physician. It is worth noting that the machine used in dataset2 was different from the one used in dataset1, and the model training was entirely based on dataset1. Therefore, dataset2 contained more noise that could have interfered with the reasoning of the model. However, the final results showed that the model successfully mitigated the interference and maintained diagnostic stability.

**Figure 4:**
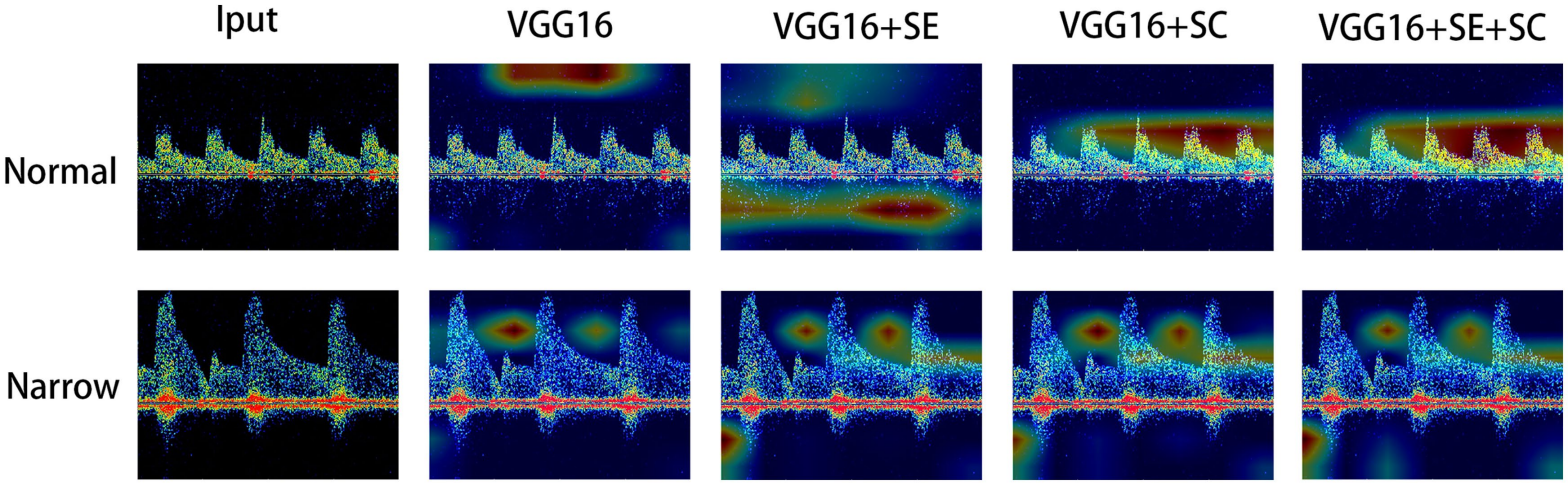
Visual thermodynamic diagram. Normal represents the normal control, while narrow represents the spectrum with stenosis in the TCD image. The color of the heat map from red to blue represents the focus of the model to identify features from strong to weak.

Finally, we have drawn a heatmap in Figure 4 to visualize the focus of feature extraction among the different models. From this, we can see that the different DL models do not consistently focus on the same areas of the TCD images. The VGG16 algorithm’s feature extraction centers deviate from the central region of the image, paying more attention to the upper region and other background areas. Due to the lack of an attention mechanism and adequate target feature learning capabilities, the algorithm’s focus deviates somewhat. The VGG16 + SE algorithm demonstrates slightly improved feature extraction capabilities compared to VGG16. It not only focuses on background information but also pays more attention to the information of blood flow pulses themselves, such as the intervals and the heights of blood flow pulses. The squeeze-and-excitation module effectively enhances sensitivity, which enables the algorithm to recognize more objects that belong to the class, paying attention to both background information and the information of blood flow pulses. The example image shows that the most concentrated areas on the heat map are around the apex of the pulse and its surrounding area. This might suggest that using network modules based on skip connections can integrate image features from different scales, enabling the model to pay closer attention to the pulse information in the images, which is a distinct feature area. This leads to more accurate recognition of target objects, while non-target objects are not as easily recognized. Overall, the VGG16 + SE + SC algorithm performs the best. It not only pays attention to the background information of the image but also focuses more on the key pulse areas in the image. As it integrates an attention mechanism and multi-scale fusion technology, it can more accurately characterize such target objects. Therefore, it can provide more accurate and reliable identification results in clinical medicine.

## 4 Discussion

In this study, we utilized and improved the VGG16 model to achieve the automatic recognition of MCA stenosis in TCD images. Subsequently, we trained this model on a dataset of TCD examination images from hospitalized patients at our hospital. Furthermore, we validated the model using a combined dataset consisting of TCD examination images from hospitalized patients (dataset1) and a population undergoing routine medical check-ups (dataset2). The results demonstrated that the enhanced VGG16 algorithm performed well on both dataset1 and dataset2. In addition, the model exhibited a high level of agreement with human physicians for dataset2, indicating a promising achievement in attaining the performance level of experienced TCD physicians (see Table 2).

**Table 2:** Performance of the different models on dataset2.

Dataset2					
Model	Accuracy (%)	Precision (%)	Specificity (%)	Sensitivity (%)	AUC
VGG16	91.48 ± 3.39	59.26 ± 15.30	93.00 ± 4.99	77.78 ± 11.11	0.8539 ± 0.031
VGG16 + SE	94.07 ± 1.70	69.97 ± 11.20	95.88 ± 2.85	77.78 ± 11.11	0.8683 ± 0.044
VGG16 + SC	87.04 ± 7.57	48.83 ± 20.82	83.54 ± 4.99	98.77 ± 2.14	0.9115 ± 0.016
VGG16 + SE + SC	93.70 ± 2.80	62.65 ± 11.27	93.00 ± 3.11	100.00 ± 0.00	0.9650 ± 0.016

TCD is one of the widely used methods in clinical medicine to detect blood flow in the cerebral arteries. It non-invasively and continuously evaluates the degree of stenosis or occlusion in the MCA, anterior cerebral artery (ACA), posterior cerebral artery (PCA), basilar artery (BA), and vertebral artery (VA). This technique can provide doctors with important information about the state of blood circulation in patients to help them make accurate diagnosis and treatment decisions (15). As posterior circulation is more tortuous and variable than anterior circulation, TCD has higher accuracy and specificity in identifying stenosis in the anterior circulation, especially MCA (16). Previous studies related to TCD and MCA stenosis often utilized features observed from prior medical experience, such as mean blood flow velocities in the MCA, pulsatility index, and resistance index (17–19). Recent studies have proposed classifying TCD image waveforms into several categories using cluster analysis, thereby investigating the diagnostic significance of different waveform types for stenosis (20). However, overall, due to the limitations of traditional analysis methods, it is often impossible to effectively integrate all features to construct a diagnostic model for TCD image abnormalities.

In recent years, the continuous development and maturation of DL technology have led to an increasing number of research reports on the significant role of DL in the diagnosis of various forms of medical imaging. For instance, Sakli et al. (21) utilized ResNet-50 for the automatic recognition and diagnosis of 12-lead electrocardiograms, achieving impressive results across multiple datasets after validation. This highlights the potential of DL in medical image diagnostics and demonstrates the feasibility of using DL models for automated medical image diagnostics. Exploring the feasibility of DL for TCD imaging then becomes particularly important.

In the field of cerebrovascular studies, DL is predominantly utilized for classifying and segmenting pathological images from MRI and CT scans. However, there is still comparatively little research on the use of DL for the recognition of TCD images. Mei YJ et al. trained an automatic TCD image classification model on a dataset consisting of 278 patients (22). The results suggested that deep learning can be employed for the identification of MCA stenosis in TCD imaging. However, due to potential limitations in the dataset size, there is room for improvement in the accuracy of these results. Nisha et al. (23) proposed a deep learning model called Self-ResAttentioNet18, which can distinguish healthy individuals from critically ill individuals. However, the TCD database that they used only contained six healthy subjects and 12 patients with known neurological critical conditions, which is relatively smaller compared to the sample size in this study. In our study, we increased the number of samples in the training dataset, which further improved the diagnostic performance of the trained model. We quantitatively compared it to the diagnostic skills of neurologists with varying levels of experience in TCD. The results showed that after the improvements were made to the VGG-16 network model, the level of diagnosis (as measured by AUC) significantly enhanced and it showed promise for future applications in TCD cerebrovascular stenosis screening. Simultaneously, we compared our results with similar studies in the same field. For instance, Mei and colleagues reported an AUC of 0.80 for their CNN model in diagnosing middle cerebral artery stenosis on the test set (22). In another similar study, researchers utilized an ensemble RNN to diagnose stenosis in 35 patients with ischemic stroke, achieving a maximum accuracy of up to 85% (24). These findings indicate that some metrics, such as AUC, of our improved model outperformed those of some existing relevant research. Moreover, no studies have been found so far that utilized DL in TCD imaging for routine physical examination populations, nor have there been comparisons of diagnostic skills between neurologists with varying years of experience. This suggests that our study fills a gap in the existing literature and may provide a valuable reference for future research in the direction of stroke screening using TCD. In addition, we believe that the agreement detected between the physician and model, tested using the kappa coefficient, will be an important preliminary step to help with TCD image classification using DL.

Regarding dataset2, although it did not directly participate in the model training, the models still maintained high accuracy in recognizing this new dataset, which demonstrates the strong generalization capability of the deep learning models. We believe there are two main reasons for this generalization ability: First, the “hospital patient dataset” was used for training and the “routine check-up population dataset” was used for testing. Although these two datasets come from different sources, their underlying data distribution patterns are fundamentally consistent. Therefore, the AI model can make a good response to the same data distribution. Second, in this study, we used domain generalization techniques based on data augmentation (25). Typical augmentation operations include scaling, cropping, color transformation, etc. They are widely used in supervised learning to improve the generalization performance of the model and reduce the occurrence of overfitting. In addition, noise addition (such as Gaussian noise) was applied on the dataset, which is an effective domain randomization method that perturbs the features, ultimately achieving good results in validation across different datasets (26). For instance, considering the transformation of the image color space from RGB to HSV, the conversion formula is as follows:


$$V=\max\left(\mathrm{R,G,B}\right)$$

$$S=\{\begin{array}{c} \frac{V-\min\left(RGB\right)}{V}\\ 0,\mathrm{else} \end{array},\;V\neq \;0$$

$$H=\{\begin{array}{c} \frac{60\left(G-B\right)}{V-\min\left(\mathrm{R,G,B}\right)},V=R\\ 120+\frac{60\left(B-R\right)}{V-\min\left(\mathrm{R,G,B}\right)},V=G\\ 240+\frac{60\left(R-G\right)}{V-\min\left(\mathrm{R,G,B}\right)},V=B \end{array}$$

After computing the value of H, another check should be performed as follows:


$$H=\{\begin{array}{c} H+360,H<0\\ H,\mathrm{else} \end{array}$$

Although dataset 2 used in this study had limited positive cases, suggesting a potential data deviation, it reflects the realistic scenario, as we aimed to simulate the effectiveness of DL in screening for cerebral artery stenosis in real-world TCD examinations. The experimental results indicate that our improved DL model achieved extremely high sensitivity in identifying typical cerebral artery stenosis in the population undergoing routine medical check-ups. In the current medical landscape, TCD reports are routinely assessed by senior physicians, which increases the demand for medical education costs and corresponding resources. We propose that our model can be progressively integrated into the report review system as an auxiliary evaluation metric, thereby alleviating these burdens. Furthermore, we will consider exploring the possibility of combining it with medical multimodal large language models to enhance its role in future medical scenarios (27). These integrations would not only optimize reporting efficiency but also potentially enhance the accuracy of the reports, ultimately benefiting a larger number of patients. It is believed that in the future, this model has the potential to significantly enhance the detection rate and accuracy of ICAS in primary healthcare facilities. Furthermore, considering the urgent need for ICAS screening in rural or underdeveloped areas, and comparing it with the high costs and maintenance expenses of MRI equipment, utilizing AI-assisted TCD examinations undoubtedly presents an optimal solution. On the other hand, before promoting this method for clinical application, we still need to carefully evaluate the demand for AI for computing resources and its potential impact on patient privacy and data security. In the current research field strategies involving federated learning and edge computing are widely adopted to address these issues. These methods not only significantly reduce the reliance on centralized computing resources but also ensure the security and effective utilization of data while fully protecting privacy (28, 29). Given these advantages, we plan to further explore and apply these advanced technological strategies in our future research.

In addition, in this study, we observed that the number of features used by the AI for diagnosing MCA stenosis from TCD exceeded one hundred. In contrast, neurologists typically utilize only a few to about a dozen diagnostic features. Due to the inherent “black box” phenomenon of deep learning (30), we are currently unable to fully interpret which features of the TCD the AI employs. Further research is needed to clarify the logic behind AI diagnoses, and we hope that this will not only enhance the diagnostic performance of AI but also provide new insights and inspiration for clinical medical practitioners regarding diagnostic approaches.

This study has certain limitations. For instance, the number of physical examination populations, also known as dataset 2, was relatively small. Due to the nature of the physical examination population, the proportion of ICAS cases in this dataset was significantly lower than that in dataset 1. Moreover, the physical examination population had fewer complex hemodynamic conditions compared to the hospitalized patients, which might have resulted in a relatively simpler distribution of the TCD images. This might have introduced biases in assessing the generalization ability of the model. These biases could have led to unexpectedly better performance of the model in dataset 2 than in dataset 1. In addition, dataset2 contained very few extreme cases, such as severe stenosis and occlusion, which is not conducive to a comprehensive evaluation of the model. Therefore, we plan to collect data from a larger-scale physical examination population in future studies to ensure a more objective evaluation of our model. We look forward to further improving the reliability of our model in future multi-center data studies so that it can play a significant role in screening for ICAS.

## Data Availability

The raw data supporting the conclusions of this article will be made available by the authors, without undue reservation.
